# Analysis of Gait Characteristics Using Hip-Knee Cyclograms in Patients with Hemiplegic Stroke

**DOI:** 10.3390/s21227685

**Published:** 2021-11-19

**Authors:** Ho Seok Lee, Hokyoung Ryu, Shi-Uk Lee, Jae-sung Cho, Sungmin You, Jae Hyeon Park, Seong-Ho Jang

**Affiliations:** 1 Department of Rehabilitation Medicine, Hanyang University College of Medicine, 222-1, Wangsimni-ro, Seongdong-gu, Seoul 04763, Korea; skyworlds@hanyang.ac.kr; 2 Department of Technology Management, Hanyang University, 222, Wangsimni-ro, Seongdong-gu, Seoul 04763, Korea; hryu@hanyang.ac.kr; 3 Department of Rehabilitation Medicine, Seoul National University College of Medicine, SMG-SNU Boramae Medical Center, 20 Boramae-ro 5-gil, Dongjak-gu, Seoul 07061, Korea; shiuk.lee@gmail.com; 4 Korea Orthopedics & Rehabilitation Engineering Center, 26, Gyeongin-ro 10beon-gil, Bupyeong-gu, Incheon 21417, Korea; jscho@kcomwel.or.kr; 5 Department of Biomedical Engineering, Hanyang University, 222, Wangsimni-ro, Seongdong-gu, Seoul 04763, Korea; seungmin@hanyang.ac.kr; 6 Department of Rehabilitation Medicine, Hanyang University Guri Hospital, 153, Gyeongchun-ro, Guri-si 11923, Korea

**Keywords:** stroke, gait disorder, hip-knee cyclogram, gait analysis

## Abstract

Gait disturbance is a common sequela of stroke. Conventional gait analysis has limitations in simultaneously assessing multiple joints. Therefore, we investigated the gait characteristics in stroke patients using hip-knee cyclograms, which have the advantage of simultaneously visualizing the gait kinematics of multiple joints. Stroke patients (*n* = 47) were categorized into two groups according to stroke severity, and healthy controls (*n* = 32) were recruited. An inertial measurement unit sensor-based gait analysis system, which requires placing seven sensors on the dorsum of both feet, the shafts of both tibias, the middle of both femurs, and the lower abdomen, was used for the gait analysis. Then, the hip-knee cyclogram parameters (range of motion, perimeter, and area) were obtained from the collected data. The coefficient of variance of the cyclogram parameters was obtained to evaluate gait variability. The cyclogram parameters differed between the stroke patients and healthy controls, and differences according to stroke severity were also observed. The gait variability parameters mainly differed in patients with more severe stroke, and specific visualized gait patterns of stroke patients were obtained through cyclograms. In conclusion, the hip-knee cyclograms, which show inter-joint coordination and visualized gait cycle in stroke patients, are clinically significant.

## 1. Introduction

Stroke is one of the main causes of long-term disability, including gait disturbances [1]. Gait disturbance in stroke patients can impair their quality of life and increase their risk of falls [2,3]. Two main factors that can lead to gait disturbance in patients with hemiplegic stroke are weakness due to damage to the motor cortex or corticospinal tract and spasticity due to a loss of supraspinal inhibition [3].

To evaluate gait patterns in stroke patients, previous studies have conducted conventional gait analysis to investigate the differences between hemiplegic gait and normal gait [4,5,6,7,8]. Mat et al. reported smaller angular excursions, longer stance phases, shorter stride length, and slower gait speed in hemiplegic gait compared to those in normal gait [9]. Reduced hip and knee joint angles in the sagittal plane of hemiplegic gait have also been reported [10]. Gait velocity is another parameter widely used to analyze the overall gait performance of stroke patients, which tends to be lower than that of healthy people [2,7,11]. However, the results of conventional gait analysis, which provide information on many parameters separately, pose a challenge in assessing multiple joints simultaneously. Since gait is the result of cooperative movement of the hip, knee, and ankle joints, a problem in one joint can affect another, changing the entire gait cycle. This implies that by assessing multiple joints simultaneously, we can analyze the conjoint motions of multiple joints more easily. Therefore, other methods that can integrate the kinematics of multiple joints in hemiplegic gait are needed.

Cyclograms in gait analysis are closed trajectories obtained by plotting one joint angle versus other joint angles to reflect gait kinematics throughout the entire gait cycle [12]. For instance, hip-knee cyclograms obtained by plotting the hip joint angle versus the knee joint angle can show the relationship between these two joint angles. Thus, cyclograms can provide information regarding inter-joint coordination and the relationships between multiple joints. Cyclograms were previously used for analyzing gait kinematics; focusing on boundary, area, and shape of the closed loop of a cyclogram can provide an intuitive geometric assessment of the gait cycle and information regarding conjoint angular movements of multiple joints [12,13,14]. Cyclograms may have advantages over conventional gait analysis in patients with stroke, because abnormal gait patterns are usually observed in multiple joints rather than in a single joint in these patients. However, only a few studies have reported the gait characteristics of patients with stroke based on cyclograms. Mah et al. classified stroke patients according to the degree of gait function and showed gait kinematics by correlating gait function with cyclograms [9]. Pilkar et al. reported on the joint symmetry of post-stroke patients based on cyclograms [15]. However, these studies included relatively small groups of participants. Moreover, no previous studies using hip-knee cyclograms have visualized cyclograms representing specific gait patterns of the stroke patients or shown differences in gait characteristics according to stroke severity.

Therefore, this study investigated the gait characteristics of patients with hemiplegic stroke according to stroke severity compared with age-matched healthy controls based on the analysis of hip-knee cyclograms. We hypothesized that the inter-joint coordination and gait variability in stroke patients would differ from those in the control group and that the specific characteristics of gait patterns in stroke patients could be represented geometrically by hip-knee cyclograms.

## 2. Materials and Methods

### 2.1. Participants

This case-control study was approved by the Institutional Review Board of the University Hospital (IRB File No. 2020-11-006). All participants provided written informed consent. The inclusion criteria were (1) hemiplegic stroke, (2) ability to walk alone or with some physical assistance, and (3) age 19 years or older. The exclusion criteria were (1) functional ambulation categories (FAC) score 0 or 1, (2) any uncontrolled medical diseases, (3) disability of gait or posture before stroke due to psychological or neurological disorders, (4) cognitive disorders that could limit the ability to understand the investigator’s instructions, (5) history of seizure except for febrile convulsions, and (6) any other problems that could limit participation in this study. For the control group, healthy people with no history of diagnosed stroke or other medical problems and no difficulty in walking were recruited. We categorized hemiplegic participants using the FAC score [8,16]. Participants in the mild stroke group had FAC scores of 4 or 5 and were able to walk independently, while participants in the moderate stroke group had FAC scores of 2 or 3 and were able to walk with physical assistance or supervision. Patients with severe stroke (FAC score 0 or 1) were excluded because they could not walk or could only walk with continuous support [16].

### 2.2. Gait Analysis

**Figure 1:**
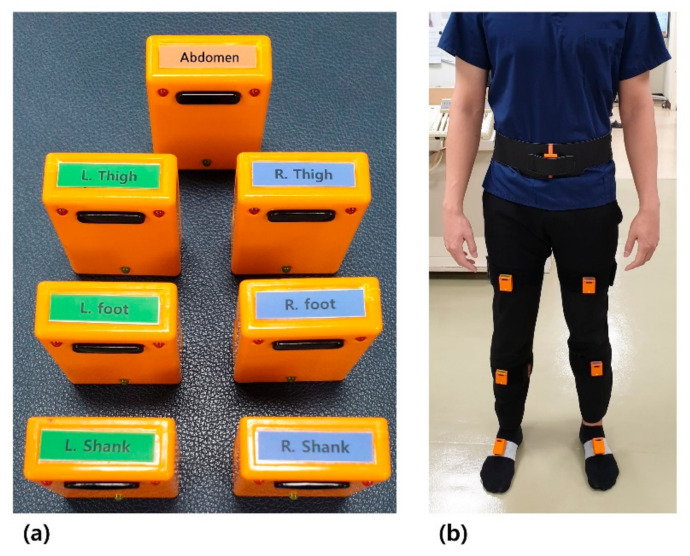
(**a**) The IMU sensors used in gait-analysis system; (**b**) The IMU sensors placed on the. dorsum of both feet, the shafts of both tibias, the middle of both femurs, and the lower abdomen.

We used an inertial measurement unit (IMU) sensor-based gait analysis system (Human Track, R. Biotech Co. Ltd., Seoul, Korea), which has been used in previous studies [14,17,18]. The accuracy of this system has been validated against the widely used three-dimensional gait motion analysis system proposed by Cho et al. [17]. Similarly, Qiu et al. demonstrated the effectiveness of the IMU using an IMU sensor-based motion capture system that significantly reflects the actual gait [19]. Sun et al. showed good recognition performance for elderly gait using an IMU-based wearable sensor, and Celik et al. also demonstrated the effectiveness of IMU-based wearables in gait analysis combined with electromyography data [20,21]. The IMU sensors consisted of a gyroscope, accelerometer, and magnetometer sensors, and all signals were collected at a frequency of 100 Hz. A total of seven IMU sensors were placed on the dorsum of both feet, the shafts of both tibias, the middle of both femurs, and the lower abdomen, as shown in Figure 1. After proper placement of the IMU sensors, all participants completed a 10 m gait course at their own gait speed. The 10 m gait course was designed to walk in a straight line, looking forward on a flat ground. All signals from each sensor were collected and the temporospatial, kinetic, and kinematic parameters of participants’ gait were analyzed. The errors that could be measured with the sensors were minimized by calibrated measurements using sensor bias and gain, which were previously validated [22,23,24].

**Figure 2:**
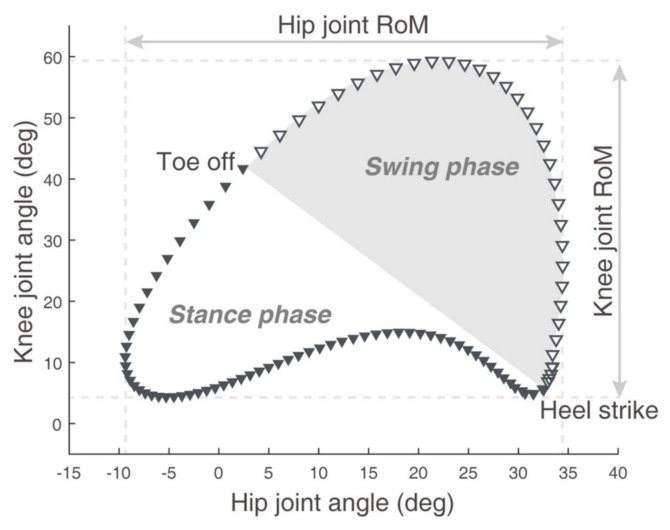
A representative sagittal plane hip–knee cyclogram. Hip and knee joint angles (degrees) during the gait cycle are plotted in the clockwise direction on the X– and Y–axes, respectively. The gait cycle is divided into stance (filled inverted triangles) and swing (open inverted triangle) phases. RoM = range of motion. This “Figure 2” by Park et al., is licensed under CC BY 4.0, modified from the original [14].

After acquiring the gait parameters, hip-knee cyclograms were obtained by simultaneously plotting the hip and knee joint angles on one plane throughout the entire gait cycle [12,25]. The hip–knee cyclograms used in this study were plotted in a clockwise direction, from the stance phase, which was calculated from the heel strike point to the toe-off point and to the swing phase, which was calculated from the toe-off point to the heel strike point, as described previously [14]. Then, we obtained the average value of hip-knee cyclogram parameters including the range of motion (ROM) of the hip and knee joint angles, perimeters, and areas. A representative hip-knee cyclogram used in this study is shown in Figure 2.

We also calculated the coefficient variances (CV) of the hip-knee cyclogram parameters for gait variability analysis [14,26,27,28] using the following equations:

(1) $$CV_{i,p}=\frac{\sigma_{i,p}}{{\bar{x}}_{i,p}}$$

(2) $${\mathrm{CV}}_{p}=\frac{\sigma_{p}}{{\bar{x}}_{p}}$$

(*i* = each participant, *p* = parameter (ROM, perimeter, and area), *σ* = standard deviation, $\bar{x}$ = the mean of parameter (ROM, perimeter, and area) [14]. The CV_*i*,*p*_ is the CV of each participant for the parameter. The CV_*p*_ is the CV of all participants for the parameter, which represents the gait variability.

The perimeter was calculated as the linear summation of the lengths of the lines connecting the data points that consisted of hip-knee cyclograms. The area was calculated as the space enclosed by the perimeter. The perimeter and area were calculated using the following equations:

(3) $$L_{i}=\sqrt{{\left(\theta_{h_{i}}-\theta_{h_{i+1}}\right)}^{2}+{\left(\theta_{k_{i}}-\theta_{k_{i+1}}\right)}^{2}}=\Delta t\sqrt{\omega_{h_{i}}{}^{2}+\omega_{k_{i}}{}^{2}}$$

(4) $$L=\sum_{i}L_{i}$$

(5) $$\mathrm{Area}=\frac{1}{2}\sum_{i}(\theta_{h_{i}}\theta_{k_{i+1}}-\theta_{h_{i}+1}\theta_{k_{i}})$$

($\theta_{h_{i}}$ and $\theta_{k_{i}}$ represent the hip and knee joint angles, respectively, of point *i*. $\omega_{h_{i}}$ and $\omega_{k_{i}}$ represent the average angular velocities of the hip and knee joints, respectively, at a specific time interval (Δ*t*)). The perimeter *L* was calculated by the summation of *L_i_*, which reflects the average joint velocity and the distance travelled by the hip and knee joints. Therefore, the perimeter could also represent the coordination between the hip and knee joints involved in the gait cycle. The area surrounded by the perimeter, which was calculated using Equation (5), represents the conjoint range of angular motion of the hip and knee joints [12,13,14,29]

### 2.3. Statistical Analysis

Kruskal–Wallis and Fisher’s exact tests were used to compare the differences in continuous and categorical variables between groups, respectively. *p*-values < 0.05, were considered statistically significant. Parameters that showed statistical significance between groups were further analyzed by multiple comparisons using Mann–Whitney U- tests with Bonferroni correction, by adjusted *p*-value (*p* < 0.017 = 0.05/3). As this study is a part of the project to validate the IMU sensor-based gait analysis system and machine learning methods on ambulatory function in patients with stroke, the sample size was not determined. The total sample size of the project was 800 participants. IBM SPSS Statistics for Windows, version 24.0 (IBM Corp., Armonk, NY, USA) was used to perform the statistical analyses.

## 3. Results

### 3.1. Participants

A total of 47 stroke patients and 32 healthy age-matched participants were recruited. Among stroke patients, 18 and 29 patients were assigned to the mild and moderate stroke groups, respectively. The demographics and clinical characteristics of all participants are shown in Table 1. There were no significant differences in age, sex, height, weight, and body mass index (BMI) between the groups. Duration, which was defined in this study as the time from the onset of stroke to the date of gait analysis, also did not differ significantly between the mild and moderate stroke groups. One participant in the mild stroke group and seven participants in the moderate stroke group wore ankle-foot orthoses (AFOs), with no significant difference between the groups. Only gait speed differed significantly between groups.

### 3.2. Hip-Knee Cyclogram Parameters

**Figure 3:**
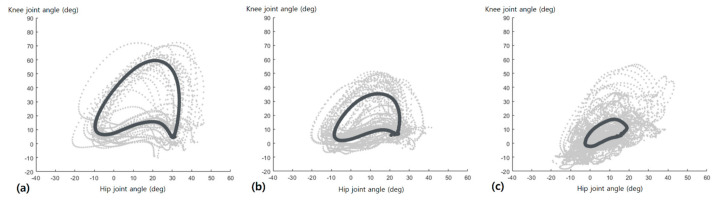
Hip–knee cyclograms of three groups. The mean hip–knee cyclogram is indicated by bold lines, while all hip–knee cyclograms are shown as gray dots. (**a**) Control group; (**b**) Mild stroke group; (**c**) Moderate stroke group.

**Figure 4:**
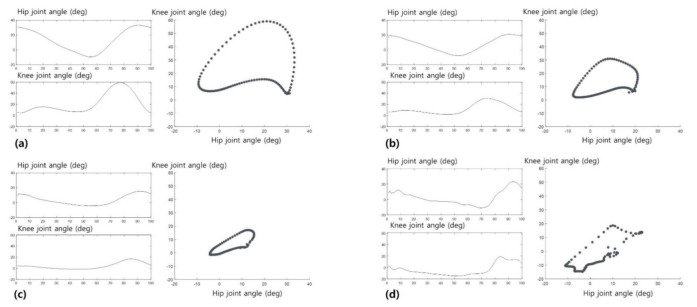
Representative hip–knee cyclograms of three groups according to the hip and knee joint ROM. (**a**) Representative from control group; (**b**) Representative from mild stroke group; (**c**) Representative from moderate stroke group; (**d**) Representative from moderate stroke group, describing hyperextension of the knee joint through the stance phase.

The mean and all hip-knee cyclograms of the three groups are shown in Figure 3. The representative hip-knee cyclograms are shown in Figure 4a–c, revealing how the cyclograms change as the hip and knee joint ROM changes. The vertical axis of the hip and knee joint ROM represents the flexion or extension degree of the joint. The horizontal axis of the hip and knee joint ROM represents a single gait cycle, which was normalized from 0 to 100 points. In addition, Figure 4d shows the representative cyclogram of participants showing hyperextension of the knee joint during the stance phase. The differences in the hip-knee cyclogram parameters of the three groups are shown in Table 2. All hip-knee cyclogram parameters showed significant differences among the three groups. Additionally, the hip-knee cyclogram parameters significantly decreased according to stroke severity, except for the stance phase area parameter. In the stance phase area, only the moderate stroke group showed significantly reduced results compared to the other groups.

### 3.3. The Coefficient of Variance (CV) for Hip-Knee Cyclogram Parameters

The results of our evaluation of gait variability using the CV of hip-knee cyclogram parameters are shown in Table 3. The CV of the total perimeter and total area differed significantly according to the stroke severity. Statistically significant increases in the CV of hip joint ROM, knee joint ROM, stance phase perimeter, and swing phase area were observed only in the moderate stroke group. The CV of the swing phase perimeter was significantly increased in both mild and moderate stroke groups compared to the control group, while no significant differences were observed between the mild and moderate stroke groups. The CV of the stance phase area did not differ significantly between the groups.

## 4. Discussion

The results of the present study identified the gait characteristics of stroke patients measured by hip-knee cyclograms and the differences in inter-joint coordination and gait variability in stroke patients compared to healthy controls. We observed differences in hip-knee cyclogram parameters according to stroke severity, except for the stance phase area, which decreased significantly only in the moderate stroke group. Within gait variability, only the CV of the total perimeter and total area increased significantly according to stroke severity. The CV of hip joint ROM and swing phase area increased significantly only in the moderate stroke group, while no differences were observed between the control and mild stroke groups. The CV of knee joint ROM, stance phase perimeter, and swing phase perimeter were significantly increased in the moderate stroke group. Notably, the CV of the stance phase area was the only parameter that did not differ significantly between the groups.

Previous studies that analyzed gait in stroke patients using traditional gait parameters showed reduced ROM of the hip and knee joint angles compared to those in healthy people [2,9,10,30]. Our study also showed a consistent reduction of hip and knee joint ROM among stroke patients compared to those in controls. We also observed significantly decreased hip and knee joint ROM according to stroke severity. The limited hip joint ROM in post-stroke patients occurs due to decreased hip flexion at heel-strike and decreased hip extension at toe-off, while the limited knee joint ROM occurs due to increased knee flexion at heel-strike and decreased knee flexion, including knee hyperextension, toe-off, and overall stance phase [2,9,10]. Therefore, as the hip and knee joint movements were more limited with more severe stroke, the tendency for decreased hip joint and knee joint ROM in our study is consistent with previous reports.

The perimeter (all stance phase, swing phase, and total), swing phase area, and total area, which represent the inter-joint coordination by the hip-knee cyclogram, also differed significantly between the groups according to stroke severity. These results may originate from the characteristics of the perimeter and area, which reflect the angular movement of the hip and knee joints [12,31]. These parameters decreased as the ROM of the hip and knee joints decreased.

However, in the stance phase area, only the moderate stroke group showed significantly decreased results compared to the other groups. As the stance phase is defined from the heel-strike to the toe-off, hip joint ROM could be more affected in the stance phase than in the swing phase in stroke patients, with more decreased hip extension in more severe stroke patients [2,32]. Within knee joint ROM, hyperextension of the knee and decreased knee flexion at toe-off were more prominent in patients with severe stroke. Knee joint hyperextension may be caused by weakness of the lower limb muscles, spasticity, and proprioceptive disabilities, which occur more frequently in patients with severe stroke [33,34]. The significant reduction in the stance phase area in the patients with more severe stroke might be due to the combination of these characteristics of the hip and knee joints. This could be a clinically significant parameter for classifying patients with mild and moderate stroke.

Furthermore, this tendency was also observed in the hip-knee cyclogram geometry shown in Figure 3. In the swing phase area, the hip-knee cyclogram showed an inverted U-shape tendency in all groups. However, in the stance phase area, the inverted U-shape of the control group generated from the heel-strike point to the toe-off point was maintained in the mild stroke group but not in the moderate stroke group. Considering the knee joint angle axis of the hip-knee cyclogram, decreased knee joint flexion during all stance phases and hyperextension in the late-stance phase are shown in Figure 3c. To our knowledge, this is the first study to show the specific geometric characteristics by the shape of hip-knee cyclograms in stroke patients according to stroke severity, and their potential as novel indicators to classify stroke severity in future studies. In addition, Figure 4 shows how the hip-knee cyclograms are presented according to the hip and knee joint ROM. As the hip and knee ROM increased, the shape of the cyclograms showed a more conjoint range of angular movements. Within the knee joint, more knee flexion in the early stance phase resulted in a more inverted U-shape in the cyclogram. Furthermore, the smoother contour of the cyclograms implies better coordinated inter-joint movement [12,13]. It may be more intuitive and easier to understand the gait cycle using cyclograms than by conventional gait analysis, which provides hip and knee joint ROM separately.

In a previous study employing cyclograms, Mah et al. reported the progression of gait pattern kinematics in patients with hemiplegic stroke [9]. Hip-knee cyclograms also changed with improvement in gait patterns. However, that study did not analyze the hip-knee cyclogram parameters (perimeter or area) and differences in those parameters according to stroke severity. In the present study, we investigated hip-knee cyclogram parameters and showed distinctive patterns according to stroke severity.

Among gait variability parameters, the CV of the total perimeter and total area showed a significantly increasing tendency according to stroke severity. Balasubramanian et al. also showed increased gait variability in stroke patients compared to controls based on the CV of traditional gait parameters [26]. Thus, the results of this study are consistent with those of a previous study. In the CV of knee joint ROM, only the moderate stroke group showed significant increases, despite the increasing tendency according to the severity. This result may be due to knee joint hyperextension in the stance phase in patients with more severe stroke [33]. The CV of hip joint ROM and the swing phase area significantly increased in the moderate stroke group compared to other groups, while there were no statistical differences between the control and mild stroke groups. These results could be due to circumduction gait or hip hiking in patients with more severe stroke [2,5,35,36]. The CV of the stance phase was the only parameter that did not differ significantly between the groups. This may be due to the significant reduction in the stance phase area in the moderate stroke group, leading to reduced variability.

The clinical significance of this study using cyclograms lies in the visual expression of gait, which shows the changes in various parameters over time. In the medical field, the visualization of numerical values using a graph can provide clinicians with an intuitive understanding. Conventional gait analysis can provide simple numerical values, such as gait speed or stride length, despite the complexity of gait. It may be easier to understand the characteristics of gait achieved by the coordination of the two joints simultaneously. Additionally, cyclograms can be used to assess the changes in gait patterns in patients with stroke more easily by changes in graphs [9]. However, conventional gait analysis may require more complicated processes to detect changes in gait patterns in patients. For example, as shown in Figure 4d, a U-shape in the stance phase of the cyclogram rather than an inverted U shape, indicates that hyperextension of the knee during the stance phase can be easily recognized by clinicians. Then, clinicians could apply specific gait training or orthosis for hyperextension of the knee joint to the patient. In this study, significant findings were obtained by hip-knee cyclograms using the IMU-based gait analysis system, which is relatively simple and cost-effective [17,19]. However, few studies have been conducted using hip-knee cyclograms; therefore, further studies are needed to assess the practical usefulness of the hip-knee cyclogram parameters shown in this study.

This study has some limitations. First, we did not consider characteristics of stroke patients, such as the stroke type and lesion location or size. Second, gait speed was not controlled during the gait analysis. Third, this study analyzed only the sagittal plane of the hip-knee cyclograms. Cyclograms in other planes (frontal or transverse), or using the ankle joint, were not obtained. Fourth, the effects of orthoses on hip-knee cyclograms were not evaluated. Previous studies have shown that AFOs mainly affect the ankle joint ROM. Furthermore, they have no significant effect on the hip joint ROM and have a minor effect on the knee joint ROM at toe-off [37,38]. In addition, participants who used an AFO wore it routinely and performed gait training with the AFO. Therefore, we conducted gait analysis to maintain the participants’ AFO use to obtain their usual gait patterns. Finally, as this was a part of the project to validate the IMU sensor-based gait analysis and machine learning methods on ambulatory function in patients with stroke, we did not conduct sample size estimation.

## 5. Conclusions

In conclusion, the hip-knee cyclograms reflect the severity of ambulatory function in stroke patients and can be used to demonstrate inter-joint coordination and gait variability based on the parameters of a cyclogram. Furthermore, the specific gait patterns of patients with stroke can be visualized intuitively and analyzed individually using hip-knee cyclograms to provide personalized gait training.

## Figures and Tables

**Table 1:** Demographic data. The results are presented as mean ± standard deviation, or *n*. *p*-values were calculated by Kruskal–Wallis and Fisher’s exact tests.

	Controls (*n* = 32)	Mild Stroke (*n* = 18)	Moderate Stroke (*n* = 29)	*p*-Value
Age (years)	63.81±7.8	63.27 ± 15.1	63.25 ± 13.1	N.S.
Sex (M:F)	14:18	10:8	13:16	N.S.
Height (cm)	162.8 ± 6.3	164.1 ± 8.4	163.4 ± 7.3	N.S.
Weight (kg)	64.0 ± 6.8	64.74 ± 9.9	63.35 ± 10.9	N.S.
BMI (kg/m^2^)	24.08 ± 2.8	23.94 ± 3.6	23.62 ± 3.2	N.S.
FAC score		4 (*n* = 10) or 5 (*n* = 8)	2 (*n* = 9) or 3 (*n* = 20)	
Gait speed (m/s)	1.33 ± 0.2	1.04 ± 0.3	0.67 ± 0.4	<0.0001
Duration (days)		215.11 ± 153.64	299.21 ± 227.95	N.S.
Orthosis (n)		1	7	N.S.

Abbreviations: *n* = number of participants; M = male; F = female; BMI = body mass index; FAC = functional ambulation categories; N.S. = not significant.

**Table 2:** Hip-knee cyclogram parameters. The results are presented as mean ± standard deviation, or *n*. *p*-values were calculated by Kruskal–Wallis tests. Multiple comparisons between groups were calculated by Mann–Whitney U-tests with Bonferroni correction.

	Controls (*n* = 32)	Mild Stroke (*n* = 18)	Moderate Stroke (*n* = 29)	*p*-Value	Multiple Comparisons		
					Controls vs. Mild	Control vs. Moderate	Mild vs. Moderate
**ROM (deg)**							
Hip	45.51 ± 5.92	38.82 ± 6.09	27.75 ± 9.45	<0.0001	<0.0001	<0.0001	<0.0001
Knee	57.43 ± 8.31	40.62 ± 7.50	27.75 ± 12.02	<0.0001	<0.0001	<0.0001	<0.0001
**Perimeter (deg)**							
Stance phase	73.05 ± 12.20	61.62 ± 13.25	49.13 ± 15.42	<0.0001	<0.0001	<0.0001	<0.0001
Swing phase	112.47 ± 12.76	82.40 ± 16.75	62.58 ± 25.00	<0.0001	<0.0001	<0.0001	<0.0001
Total	185.52 ± 21.93	144.03 ± 20.88	111.72 ± 33.58	<0.0001	<0.0001	<0.0001	<0.0001
**Area (deg^2^)**							
Stance phase	213.10 ± 170.03	212.13 ± 212.27	86.36 ± 92.94	<0.0001	N.S.	<0.0001	0.0002
Swing phase	1468.67 ± 345.46	743.68 ± 274.92	277.24 ± 326.90	<0.0001	<0.0001	<0.0001	<0.0001
Total	1609.70 ± 431.78	895.30 ± 375.19	335.80 ± 364.28	<0.0001	<0.0001	<0.0001	<0.0001

Abbreviations: *n* = number of participants; ROM = range of motion; deg = degree; N.S = not significant.

**Table 3:** The coefficient of variance (CV) for hip-knee cyclogram parameters. The results are presented as mean ± standard deviation, or *n*. *p*-values were calculated by Kruskal–Wallis tests. Multiple comparisons between groups were calculated by Mann–Whitney U-tests with Bonferroni correction.

	Controls (*n* = 32)	Mild Stroke (*n* = 18)	Moderate Stroke (*n* = 29)	*p*-Value	Multiple Comparisons		
					Controls vs. Mild	Control vs. Moderate	Mild vs. Moderate
**ROM (deg)**							
Hip	5.03 ± 3.17	5.33 ± 3.94	11.38 ± 8.25	<0.0001	N.S.	<0.0001	0.0050
Knee	4.34 ± 2.79	6.82 ± 3.68	9.79 ± 8.25	0.0011	N.S.	0.0004	N.S.
**Perimeter (deg)**							
Stance phase	6.39 ± 3.93	10.13 ± 6.16	16.79 ± 12.41	<0.0001	N.S.	<0.0001	N.S.
Swing phase	5.22 ± 2.41	10.11 ± 6.34	17.51 ± 17.40	<0.0001	0.0031	<0.0001	N.S.
Total	3.18 ± 1.85	5.37 ± 2.83	11.69 ± 11.29	<0.0001	0.0078	<0.0001	0.0021
**Area (deg^2^)**							
Stance phase	51.51 ± 25.12	53.16 ± 23.29	57.38 ± 17.31	N.S.	-	-	-
Swing phase	11.00 ± 6.86	20.09 ± 16.73	39.81 ± 27.40	<0.0001	N.S.	<0.0001	<0.0001
Total	9.55 ± 5.88	14.27 ± 5.33	34.23 ± 23.30	<0.0001	0.0020	<0.0001	0.0028

Abbreviations: *n* = number of participants; ROM = range of motion; deg = degree; N.S. = not significant.

## Data Availability

The data presented in this study are available upon request from the corresponding authors.
